# Complete mitochondrial genome of *Polyphylla gracilicornis* (Coleoptera：Scarabaeoidea)

**DOI:** 10.1080/23802359.2020.1870895

**Published:** 2021-02-11

**Authors:** Yuantao Zhou, Jingyan Yan, Hexing Qi

**Affiliations:** State Key Laboratory of Plateau Ecology and Agriculture, College of Agriculture and Animal Husbandry, Qinghai University, Xining, Qinghai, China

**Keywords:** *Polyphylla gracilicornis*, mitogenome, sequence, phylogenetic analysis

## Abstract

*Polyphylla gracilicornis* is one of the important underground pest species that damage agricultural and forestry plants and often requires chemical control during outbreak. Here, we determined the complete mitochondrial genome sequence of *P. gracilicornis* (GenBank accession no. MW143080) using Illumina NovaSe Sequencing System with a read length of 150 bp. The complete mitogenome consists of a 16,793 bp circular DNA molecule and the overall base composition was 36.97% A, 31.95% T, 10.41% G and 20.67% C. The full mitochondrial genome contains 38 sequence elements: 13 protein-coding genes (PCGs), 22 transfer RNA (tRNA) genes, two ribosomal RNA (rRNA) genes, and a putative control region (CR). All protein-coding genes of *P. gracilicornis* have the typical ATN (Met) start codons and typical TAN stop codons. Phylogenetic analysis revealed that *P. gracilicornis* clustered into a clade with homologous species with high bootstrap support.

*Polyphylla gracilicornis* is originally an underground pest that harms forest fruit trees, which with the adjustment of planting structure has been transferred to harm crops in recent years. In China, it is one of the main agricultural and forest pests in various provinces and regions in Northwest and North China (Liu et  al. 1997). The adult feed on a variety of crops and fruit tree leaves, flower buds, shoots, and young fruits. The larvae feed on the germinated seeds and bite off the roots and stems which cause uneven seedlings or destroy the nursery (Cui and Li 1996). Up to now, there are a few studies on *P. gracilicornis*, mainly focusing on life history, life habits, drug efficacy tests, Bioassay, etc. (Wei et al. 1989; Weiner et al. 1990; Hui and Turvey Nigel 1996). Also, its genetic structure and phylogenetic status have not been reported yet. In this study, *P. gracilicornis* was used as the research object. For the first time, the complete mitochondrial genome sequence was determined and compared with the reported insect mitochondrial genome information, in order to promote studies on the population genetics, evolution, and taxonomy of *P. gracilicornis* and related species.

In this study, samples of *P. gracilicornis* adult were obtained from Xining, Qinghai Province, China (36°62′N, 101°77′E) in September 2020 and stored in the Insect Collection of the Entomology Lab, College of Agriculture and Animal Husbandry, Qinghai University with Specimen Accession Number: ZYT-202009-01. We extracted genomic DNA from a single sample, then used Illumina Nova Sequencing System and (Illumina, San Diego, CA, USA) to determine the complete mitochondrial DNA sequence. Sequencing read length was 150 bp, 5.5 G raw data were finally yielded. The SPAdes v3.10.1 software (http://cab.spbu.ru/software/spades/) (Bankevich et al. 2012) was employed to assemble the mitogenome, gaps of which were filled with SSPACE 3.0 (Boetzer et al. 2011) and GapFiller 1.1 (Boetzer and Pirovano 2012). The assembled sequence was then annotated using the web server MITOS (Bernt et al. 2013).

The complete mitogenome of *P. gracilicornis* consistes of a 16,793 bp circular DNA molecule and deposited in the GenBank with accession number of MW143080. The overall base composition is 36.97% A, 31.95% T, 10.41% G, and 20.67% C. The AT-skews and GC-skews of the major strands of the mitogenome were calculated to be approximately 0.073 and −0.330, respectively. The full mitochondrial genome contains 38 sequence elements: 13 protein-coding genes (PCGs), 22 transfer RNA (tRNA) genes, 2 ribosomal RNA (rRNA) genes, and a putative control region(CR). All PCGs of *P. gracilicornis* have the typical ATN (Met) start codons for invertebrate mitochondrial PCGs, which except cox1 for TTG: one gene (cox2) – ATA; one gene (atp8) – ATC; five genes (*nad1*, *nad2*, *nad3*, *nad5*, and *nad6*) – ATT; five genes (*atp6*, *cox3*, *cob*, *nad4*, and *nad4l*) – ATG. Most of the PCGs terminate with the typical TAN stop codons: seven genes (*nad1*, *nad2*, *nad4*, *nad4l*, *nad5*, *nad6,* and *atp6*) have a TAA stop codon; three genes (*atp8*, *nad3* and *cob*) have a TAG stop codon; three genes (*cox1*, *cox2*, and *cox3*) end with the incomplete codon T that is completed by the addition of 30-A nucleotides to the resultant mRNA. The 22 tRNA genes are interspersed throughout the coding region and range from 63 to 70 bp and all the tRNA genes could be folded into a typical cloverleaf secondary structure. The lrRNA and srRNA genes are 1283 and 793 bp long, respectively. The A + T content of the whole genome, PCGs, tRNAs, and rRNAs is 68.92, 67.24, 72.33, and 72.78%.

A phylogenetic tree was constructed to analyze the phylogenetic relationship of the *P. gracilicornis* and other insects, using the maximum likelihood method in MEGA7 (Kumar et al. 2016) . The maximum likelihood method was used to create phylogenetic trees with the JTT model, uniform rates, partial deletion, nearest neighbor-interchange heuristic method and default automatic NJ/BioNJ. Bootstrapping was assessed with 1000 bootstrap replicates, and bootstrap support greater than 70% to be displayed. A total of 10 mitochondrial genome sequences assembled here or extracted from GenBank. Phylogenetic relationships of *P. gracilicornis* with 9 closely related taxa of Coleoptera including *Tribolium castaneum*, *Oedemera virescens*, *Heterocerus fenestratus*, *Monochamus alternatus*, *Damaster mirabilissimus*, *Rhopaea magnicornis*, *Cheirotonus jansoni*, *Protaetia brevitarsis* and *Prosopocoilus astacoides* (Figure 1). The phylogenetic analysis revealed that *P. gracilicornis* is clustered together into clade with *T.castaneum*, bootstrap values is sharing 100%. Overall, our study provides insight into the mitogenome of *P. gracilicornis*, which constitutes useful resource for population genetic study, and taxonomic classification efforts on this species.

**Figure 1.**
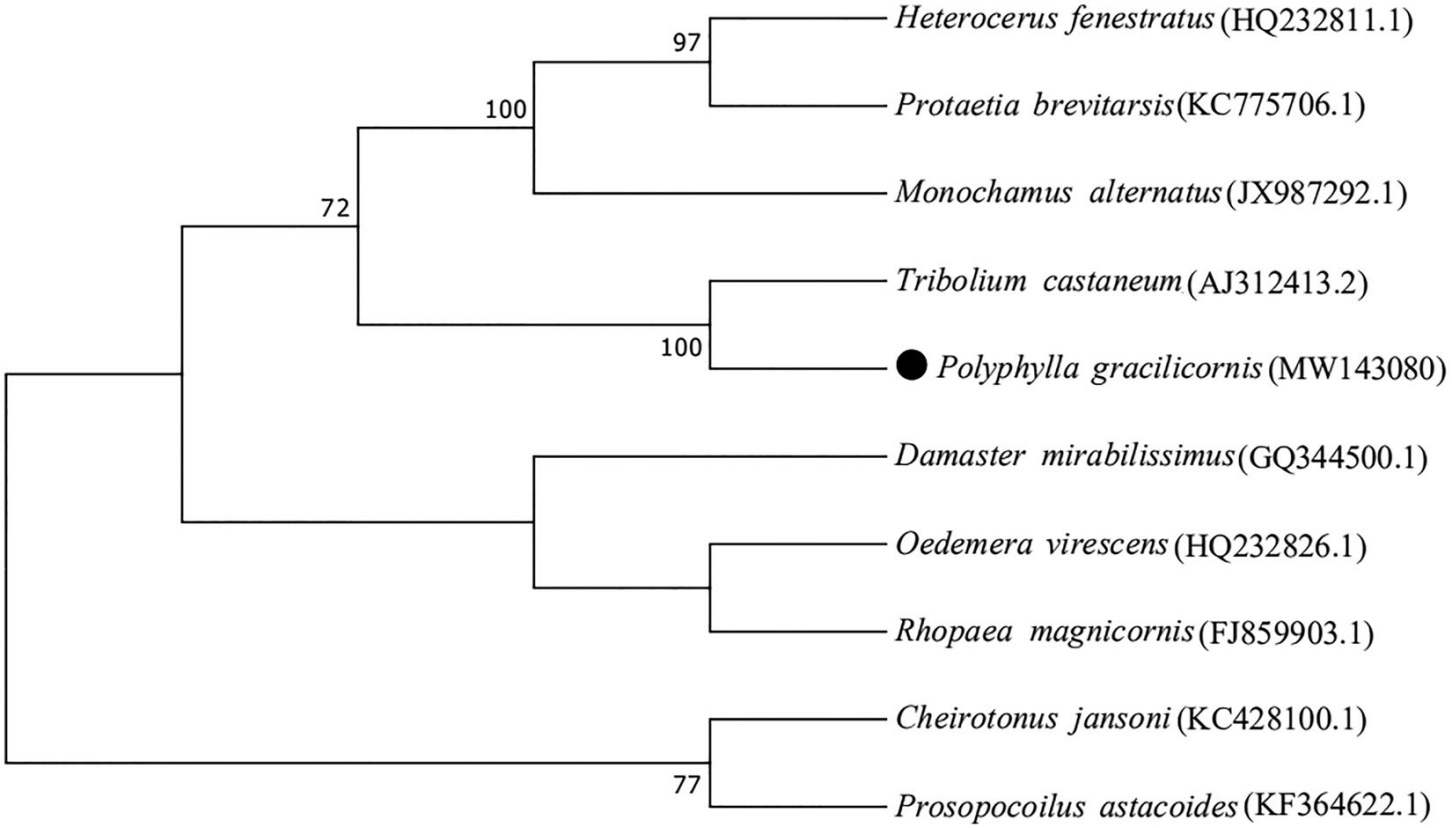
Phylogenetic tree of 10 mitochondrial genes sequences, Number above each node indicates the bootstrap support values with 1000 replicates. *Polyphylla gracilicornis* is represented by black circle.

## Data Availability

The data that is supporting the findings of this study are openly available in GenBank of National Center for Biotechnology Information at https://www.ncbi.nlm.nih.gov, reference number MW143080.
